# Malignant Gastrointestinal Neuroectodermal Tumor, a Rare Neoplasm, Presenting With Hemoperitoneum and Malena: A Case Report

**DOI:** 10.7759/cureus.70735

**Published:** 2024-10-02

**Authors:** Sampada Wankhede, Debiprasad Sahoo, Aishwarya A Meshram, Siddhesh Rane, Nitin Borle

**Affiliations:** 1 General Surgery, Topiwala National Medical College and Bai Yamunabai Laxman Nair Charitable Hospital, Mumbai, IND; 2 Gastroenterology, Topiwala National Medical College and Bai Yamunabai Laxman Nair Charitable Hospital, Mumbai, IND

**Keywords:** ascites, crohn’s disease (cd), ewsr1 rearrangement, fluorescence in situ hybridization (fish), gnet, immunohistochemistry (ihc), malena, s100

## Abstract

Malignant gastrointestinal neuroectodermal tumor (GNET) are rare malignant mesenchymal tumors. The tumor can present with various symptoms like abdominal pain, anorexia, or small bowel obstruction. Here, we present a case of small intestinal GNET who presented with gastrointestinal bleed and hemoperitoneum, a rare presentation of this disease. This patient was misdiagnosed initially as Crohn’s disease and treated for the same. However, non-response to the standard treatment and onset of new symptoms like malena and ascites raised the suspicion of some alternate diagnosis. Exploratory laparotomy showed the presence of hemoperitoneum along with a mass, 100 cm proximal to ileo-cecal junction. She was successfully treated with surgical resection and anastomosis. Histopathology, immunohistochemistry (diffuse positivity for S100 and weak positivity for synaptophysin) and molecular fluorescence in-situ hybridization (FISH) study (translocation involving the chromosomal region 2212.1-q12.2 which harbors *EWSR1* gene) confirmed the diagnosis of GNET.

## Introduction

A malignant gastrointestinal neuroectodermal tumor (GNET) is a rare malignant mesenchymal tumor. Initially, it was known as a ‘clear cell sarcoma-like gastrointestinal tumor’ (CCSLGT) [1] and was first described by Zambrano et al. in 2003 [2]. To our knowledge, only 96 cases have been reported worldwide till 2020 [3] and remains less than 100 as of now. The tumor can present with various symptoms like abdominal pain, anorexia, and small bowel obstruction. This disease does not have any specific clinical features or imaging features to suggest the diagnosis, but histopathology, immunohistochemistry, and molecular fluorescence in-situ hybridization (FISH) studies help in the definitive diagnosis of this rare disease.

Here, we present a case of a 45-year-old female who presented with unexplained severe anemia which was found to be due to gastrointestinal blood loss and hemoperitoneum caused by GNET.

## Case presentation

A 45-year-old female presented to the hospital with multiple episodes of black-colored stools and generalized weakness. She had no history of any comorbidity, no significant family history, surgical history, or allergies. The patient denied any history of addiction of medical importance.

She had past history of admission to the hospital with complaints of abdominal pain for one year, which was associated with vomiting. For this, the patient was evaluated and eventually underwent CT enterography, which was suggestive of mild aneurysmal dilatation of a short segment of proximal ileum with well-defined circumferentially enhancing wall thickening (with patent lumen) along with a single heterogeneously enhancing lymph node of size 2.5 x 2.4 cm in the mesentery just below aortic bifurcation. After this, the patient underwent enteroscopy, which showed proximal ileal stricturous narrowing and inflamed surrounding mucosa suggesting the possibility of Crohn’s disease. The patient was started on treatment for the same with azathioprine 50 mg. The patient noticed temporary symptomatic improvement. However, she had multiple episodes of black-colored stools and generalized weakness prior to her current presentation to the hospital, which made her come to the ED.

On physical examination, the patient was afebrile, heart rate was 110 beats per minute, respiratory rate was 20 breaths per minute, and blood pressure was 100/60 mmHg. The patient had pallor and the rest of the general examination was normal. Per abdominal examination, her abdomen was distended and slightly tender diffusely on palpation with an abdominal girth of 100 cm. Rectal examination showed the presence of black stools. On blood investigations patient had severe anemia, with hemoglobin of 5.8 gm/dl; rest all routine blood investigations were within normal limits, as mentioned in Table 1.

**Table 1:** Blood investigations on admission Hb: Hemoglobin; WBC: White Blood Cells; AST: Aspartate Transferase; ALT: Alanine Transferase; ALP: Alkaline Phosphatase

Investigations	Normal values	Patient values at admission
Hb	12-15 gm/dl	5.8 gm/dl
WBC	4000-11000	10200
Platelet	150-450x10^3^/microlitre	376 x 10^3^/microlitre
Total/Direct Bilirubin	0.1-1.2/0-0.2 mg%	0.7/0.14 mg%
SGOT/PT	10-40 U/L	37/22 U/L
ALP	44-147 U/L	106 U/L
Na/K	135-145/3.5-5.5 mEq/L	142/4.2 mEq/L
Serum Creatinine	0.6-1.2 mg%	1.1 mg%

**Figure 1:**
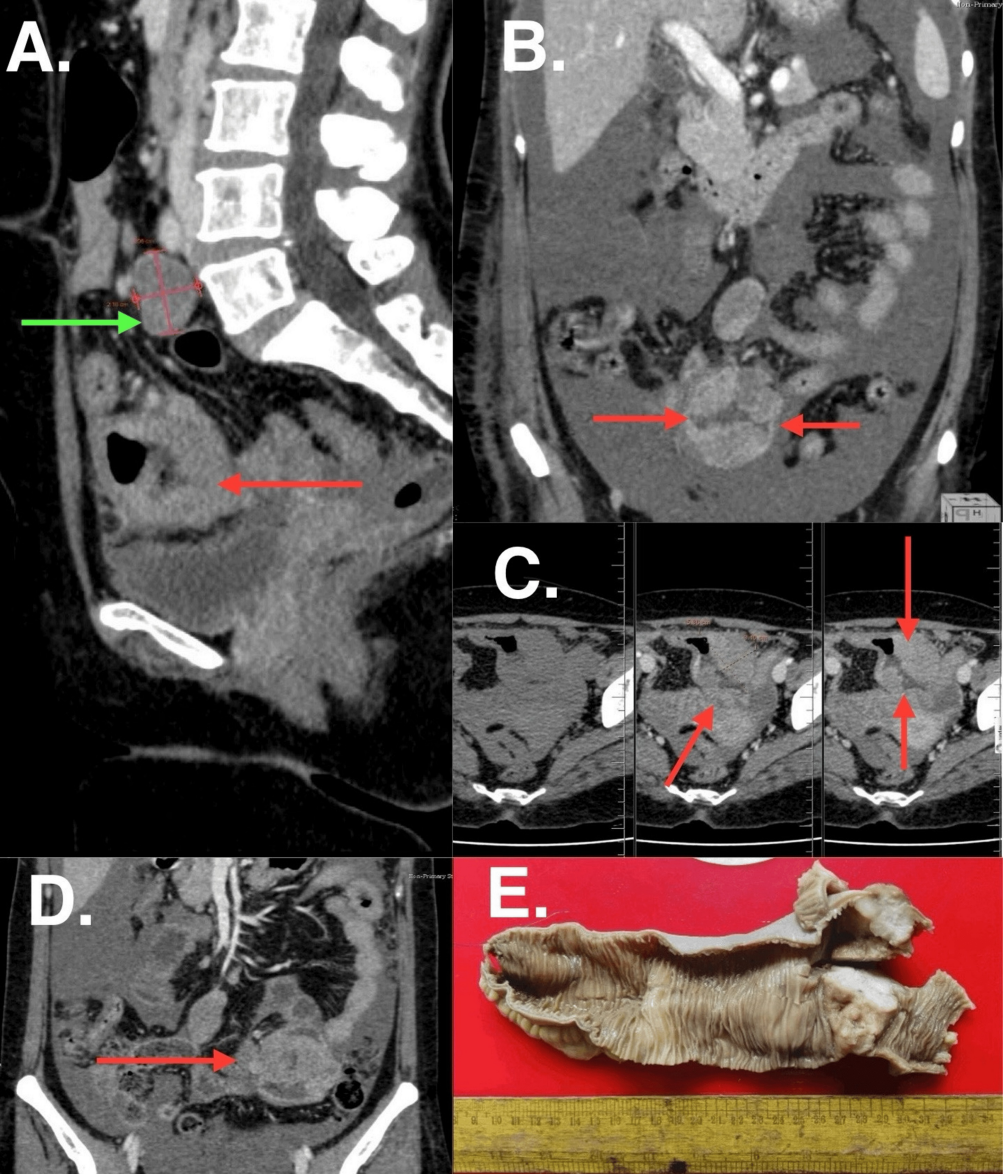
CT enterography images (A) Sagittal section, (B, D) Coronal section, (C) Axial section showing involved bowel segment (red arrow) with circumferential enhancing wall thickening; (E) Gross specimen

CT enterography was repeated this time which showed the presence of moderate ascites, in addition to mild aneurysmal dilatation of a short segment of proximal ileum with well-defined circumferentially enhancing wall thickening (with patent lumen) (Figure 1A-1D). The ascitic fluid was tapped and sent for routine microscopic examination. This showed the presence of frank blood with 3.45 gm/dl of protein, with 1.89 gm/dl of albumin, and a serum ascitic albumin gradient (SAAG) of 0.9. The total cell count was 3017 cells/mm^3^, out of which 78% were neutrophils and numerous RBCs.

After optimizing the patient and building up the hemoglobin levels, the decision was taken to perform exploratory laparotomy in view of the hemoperitoneum. Intraoperatively, a hemoperitoneum of approximately 2000 ml in volume was noticed. Also, a mass of size 3.5 × 4.5 x 2 cm at the proximal ileum, approximately 100 cm proximal to the ileocecal junction was found. Resection anastomosis of this ileal lesion was performed. The rest of the bowel appeared normal. One enlarged lymph node was also seen intraoperatively and was resected. The resected specimen on macroscopic examination showed proximal ileum with an ulcero-infiltrative lesion of size 3.5 x 4.5 x 2 cm (Figure 1E) with a proximal resection margin of 17 cm and a distal resection margin of 5 cm, free of tumor.

**Figure 2:**
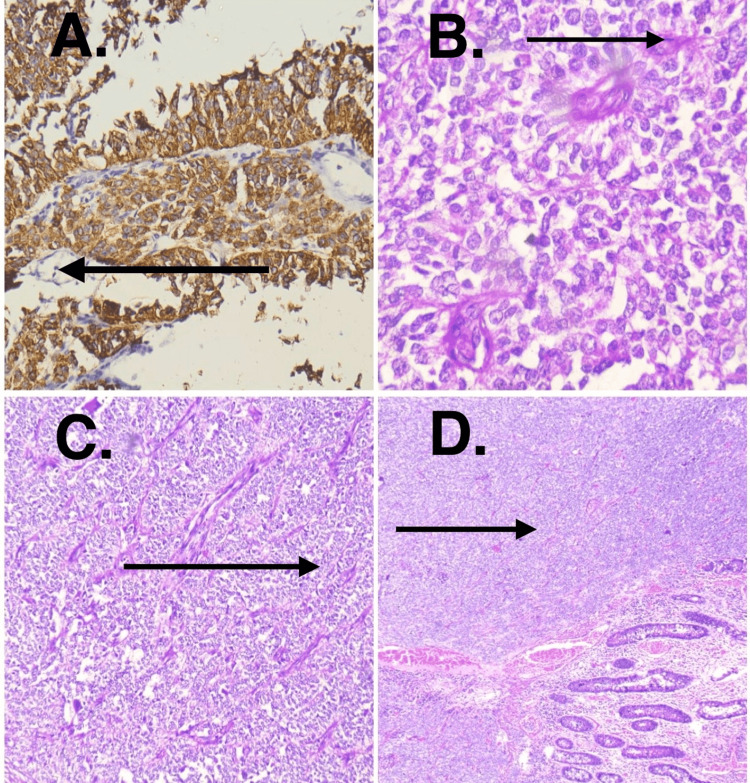
(A) Synaptophysin positivity in IHC. (B) Tumor cells arranged in sheets, clusters and rosettes; cells have vesicular to hyperchromatic nuclei with prominent nucleoli with salt and pepper chromatin. (C) Organoid, nests, and trabecular arrangement of cells. (D) Neoplastic cells in mucosa and submucosa along with normal ileal mucosa. IHC: Immunohistochemistry

Histologically, high-grade malignant tumor composed of closely packed cells with undifferentiated morphology was present. The tumor showed involvement of full thickness of the ileum along with serosa. Focally tumor cells showed clear cytoplasm and pseudo-papillary architecture. The tumor was arranged in vague nodules and confluent solid sheets. Thin arborizing vasculature and mitoses of 1-2/high power field (HPF) were noted. Necrosis was not seen. On immunohistochemistry (IHC), the tumor cells showed diffuse positivity for S100 and weak positivity for synaptophysin (Figure 2). It was negative for HMB45, MelanA, cytokeratin (AE1/AE3), and c-kit. These biopsy findings were consistent with GNET. Molecular FISH testing for *EWSR1* rearrangement was performed. It revealed the translocation involving the chromosomal region 2212.1-q12.2, which harbors *the EWSR1* gene. It showed a total of 100 tumor cell nuclei and 78% of nuclei showed split signals.

The patient showed symptomatic improvement in the postoperative period. The recovery was uneventful and the patient was discharged on postoperative day 7. The postoperative lab investigations are given in Table 2. She was asked to follow up with the medical oncologist for further chemotherapy.

**Table 2:** Postoperative blood investigations Hb: Hemoglobin; WBC: White Blood Cells; AST: Aspartate Transferase; ALT: Alanine Transferase; ALP: Alkaline Phosphatase

Investigations	Normal values	Postoperative patient values
Hb	12-15 gm/dl	10.8 gm/dl
WBC	4000-11000	9000
Platelet	150-450x10^3^/microliter	369x10^3^/microliter
Total/Direct Bilirubin	0.1-1.2/0-0.2 mg%	0.4/0.2 mg%
SGOT/PT	10-40 U/L	16/10 U/L
ALP	44-147 U/L	210 U/L
Na/K	135-145/3.5-5.5 mEq/L	131/3.4 mEq/L
Serum Creatinine	0.6-1.2 mg%	0.5 mg%

## Discussion

GNET is a rare malignant tumor that can affect any part of the gastrointestinal tract, the most common being the small intestine. It is often seen in young adults, without any preference for gender [4]. In previous studies, it has been described that the neoplastic cells may originate from autonomic primitive neural cells, with a complete lack of melanocyte differentiation [5] and, hence, lack melanin expression. However, it shows a rich presence of osteoclasts-like cells [6]. At the genetic level, these tumors are characterized by *EWSR1* gene rearrangement [7]. Here, we present a case of GNET involving the small intestine and presenting with bleeding manifestations and severe anemia, which is an unusual presentation of GNET.

Our patient was symptomatic nearly for one year but was initially misdiagnosed with Crohn’s disease. The patient was on treatment for the same with good compliance. However, the suspicion for alternate diagnosis was aroused when the patient had worsening of symptoms even on treatment. She presented with symptoms of black stools and generalized weakness. In view of gastrointestinal bleeding (malena), the patient underwent upper gastrointestinal endoscopy and colonoscopy which turned out to be normal. Hence, we focused on the possibility of bleeding from the small intestine for which we initially did CT enterography before opting for enteroscopy. This showed the presence of small aneurysmal dilatation of the proximal intestine and moderate ascites, a new finding as compared to the previous scan. In view of ascites, ultrasound-guided tapping of the fluid was done, which showed the presence of frank blood along with ascitic fluid. Based on the presence of hemoperitoneum, and CT findings, we kept gastrointestinal stromal tumor (GIST), lymphoma, or small intestinal malignancy as our differential diagnoses.

After this, we decided to go for exploratory laparotomy, wherein we found a hemoperitoneum of around 2000 ml in volume and a mass of size 3.5 x 4.5 x 2 cm over proximal ileum approximately 100 cm proximal to the ileocecal junction. The mass appeared infiltrative, ulcerative, and non-polypoidal, hence we proceeded to an en-masse surgical resection of 30 cm of proximal ileum followed by bowel anastomosis along with resection of a single enlarged mesenteric lymph node. The presence of an ulcero-infiltrative lesion along with the presence of an enlarged lymph node (which showed contrast enhancement on imaging) pointed towards a possibility of malignant etiology. Histopathological examination of this mass was consistent with GNET and the enlargement of the lymph node was found to be due to inflammation, without any malignant feature. Since, on IHC, the tumor cells showed positivity for S100 and weak positivity for synaptophysin, we went ahead with molecular FISH analysis. The interphase FISH test showed *EWSR1* gene rearrangement involving 22q12.1-q12.2 chromosome region. A total of 100 tumor cell nuclei were counted and 78% of nuclei showed split signals, indicating *EWSR1* gene rearrangement. The combination of histopathological features, IHC, and molecular study supported the diagnosis of GNET [2].

So far, less than 100 cases of GNET have been described in the literature, and to the best of our knowledge, this is the first case of GNET presenting as hemoperitoneum and gastrointestinal bleed. This shows that GNET is a very rare entity and can be considered as a differential diagnosis for GIST, Cronkhite-Canada syndrome of gastrointestinal tract (CCS-GI), adenocarcinoma, primary or metastatic melanoma, malignant lymphoma, and malignant granular cell tumor [7]. Imaging modalities, although have a limited role in the diagnosis of GNET, play a significant role in determining the extent of the disease and detecting the recurrence during follow-up [2]. However, histopathology and molecular study remain the gold standard for diagnosis of GNET [2,7,8]. Surgical excision of the involved bowel segment is the mainstay of treatment, followed by regular image monitoring [3,9,10]. Adjuvant chemotherapy or focal radiotherapy may serve to be useful for prolonging time to death. Chemotherapy includes treatment with common anti-tumor drugs such as ifosfamide, doxorubicin, crizotinib, and pazopanib [11,12].

## Conclusions

This report described the case of a female patient with GNET which is a rare entity, who presented with unusual manifestations of gastrointestinal bleeding (In the form of hemoperitoneum and malena, and was treated successfully with surgical resection. Further accumulation of cases of GNET would help elucidate etiology and risk factors along with the chronological organization of usual and unusual symptoms.
